# Comparison of a home-based (multi) systemic intervention to promoting Medication AdheRence and Self-management among kidney transplant recipients with care-as-usual: the MARS randomized controlled trial protocol

**DOI:** 10.1186/s12882-020-02008-z

**Published:** 2020-08-28

**Authors:** Denise Karin Beck, Mirjam Tielen, Marloes Rechards, Reinier Timman, Charlotte Boonstra, Josette Versteegh, Jacqueline van de Wetering, Robert Zietse, Teun van Gelder, Willem Weimar, Jan van Saase, Jan van Busschbach, Emma Kay Massey

**Affiliations:** 1 Department of Internal Medicine – Section Nephrology & Transplantation, Erasmus Medical Center, University Medical Center Rotterdam, Dr. Molewaterplein 40, 3015 Rotterdam, GD Netherlands; 2 Department of Medical Psychology and Psychotherapy, Erasmus MC, University Medical Center Rotterdam, Rotterdam, Netherlands; 3 Department of Pediatric Nephrology, Erasmus MC – Sophia Children’s Hospital, University Medical Center Rotterdam, Rotterdam, Netherlands

**Keywords:** Kidney transplantation, Adherence, Intervention, Behavior change, Self-management

## Abstract

**Background:**

After kidney transplantation non-adherence and inadequate self-management undermine clinical outcomes and quality of life. Both have been demonstrated to be substantial in all age groups. However, interventions promoting adherence and self-management among kidney transplant recipients that have proven to be effective are scarce. In this study we aim to develop and test an intervention to optimize adherence and self-management. In this article we describe the background and design of the trial entitled ‘promoting Medication AdheRence and Self-management among kidney transplant recipients’ (MARS-trial)’.

**Methods/design:**

This is a single-center, parallel arm randomized controlled trial. Nonadherent kidney transplant recipients aged 12 years or older are eligible for inclusion. Patients will be randomly assigned to either the experimental or a control group. The control group will receive care-as-usual. The experimental group will receive care-as-usual plus the MARS-intervention. The MARS-intervention is an outreaching intervention, based on the principles of (multi) systemic therapy which means involving the social network. A standardized intervention protocol is used for consistency but we will tailor the behavior change techniques used to the specific needs and determinants of each patient. The primary outcome of medication adherence will be measured using electronic monitoring. Secondary outcome measures regarding medication adherence and self-management are also assessed. Data is collected at baseline (T0), after a run-in period (T1), at six months post-baseline/end of treatment (T2) and after a six month follow-up period (T3).

**Discussion:**

We combined elements of (multi) systemic therapy and evidence-based behavior change techniques to create an outreaching and highly individualized intervention. In this trial we will investigate the impact on medication adherence and self-management after kidney transplantation.

**Trial registration:**

Netherlands Trial Register,trial number **NTR7462.** Registered 7th September 2018, https://www.trialregister.nl/trial/7264

## Background

Adherence can be defined as “the extent to which a person’s behavior corresponds with agreed recommendations from a health care provider” (WHO). Adherence to the immunosuppressive medication (IM) regimen and other lifestyle recommendations after kidney transplantation is related to better clinical outcomes [1, 2]. Non-adherence to IM, however, diminishes treatment effectiveness resulting in acute graft rejection [3, 4], chronic rejection [5] and graft failure [3, 6, 7]. These in turn directly affect survival of the graft. Adherence is one aspect of self-management. The broader concept of self-management is defined as:

“ *the ability of the individual, in conjunction with family, community, and healthcare professionals, to manage symptoms, treatments, lifestyle changes, psychosocial, cultural, and spiritual consequences of health conditions to maintain a satisfactory quality of life* [8]*”.*

Suboptimal self-management also undermines outcomes after transplantation in more indirect ways. For example, unhealthy behaviors, such as smoking, can lead to cardiovascular disease [9, 10], which is the number one cause of death among patients with a functioning kidney transplant [11]. Despite these potential negative consequences, IM non-adherence and suboptimal self-management are common among kidney transplant recipients of all age groups, but more prevalent in adolescents and young adults [12–16]. Moreover, multiple studies have shown that non-adherence to the IM regime and lifestyle recommendations occurs soon after transplantation [3, 17, 18] and increases over time [17–19]. Developing interventions that target non-adherence and suboptimal self-management has been suggested to be one way to optimize clinical outcomes [1]. However, interventions effective in promoting adherence and self-management are scarce [20–22]. In the current literature there are two main explanations for the lack of effects: limitations of the interventions; and limitations of the research methods.

Firstly, existing interventions have some limitations which may decrease their effectiveness [20, 21]. Interventions have been criticized for their one-size fits-all approach as non-adherence and suboptimal self-management can be the result of varying underlying problems. Tailoring interventions to the specific underlying problems increases the chance of them being effective. Tailoring interventions might also stimulate a higher level of intrinsic motivation for change because of their personal relevance. Another criticism is that existing interventions often target patients in isolation, for the most part due to practical reasons (e.g. interventions are provided in the hospital). Involving the social network and seeing problems in a broader social context can have multiple advantages, for example, sustaining behavioral change [23–26]. Removing practical barriers for involving the social network, such as outreaching interventions using home visits, seems promising [27–29]. Finally, many interventions focus on providing information instead of on behavior change and where behavior change techniques are used they are often not based on theory or evidence [20, 21]. Improving upon these shortcomings might enhance the effectiveness of an intervention.

Secondly, there are methodological shortcomings of the research conducted to test the effectiveness of interventions in changing clinical outcomes. In their review, Duncan et al. [22] highlighted ‘the streetlight effect’ whereby studies assessing adherence promoting interventions make one or both of the following errors:

*“the research is being conducted on the wrong set of patients (the researchers focus on a sample that is easiest to recruit but is not representative of the population that is supposed to be treated); or the intervention examines the wrong set of outcome measures (typically, this means studies that use process measures – measures that look at some aspect of the intervention’s purported mechanism of action but not at the actual outcomes)”* [22]*.*

To avoid the streetlight effect and therefore increase chances of finding an effect on adherence and transplant outcomes they recommended avoiding the use of convenience samples [22]. This should be avoided as you cannot demonstrate an effect of the intervention among patients who are already adherent. However, recruiting a non-adherent sample may be challenging as adherence promoting interventions require patients to be reachable, willing, to attend appointments, and to invest time and effort into behavior change. Outreaching interventions in the home environment may be able to decrease the burden for patients and their social network and therefore lower the threshold for participation. Furthermore, identification of non-adherent patients who may be in need of such an intervention can be difficult due to underreporting. This ‘tip of the iceberg’ phenomenon refers to the many patients we are not aware of, but who do not carry out treatment as intended. One way to avoid the inclusion of convenience samples and recruiting those in need of the intervention is to use multiple ways of assessing adherence instead of relying solely on data provided by the patients themselves.

Another methodological challenge when conducting a study on adherence is the choice of instrument to assess the outcomes. Electric Monitoring (EM) is considered to be the gold standard for research in this area [31, 32]. Nevertheless, a combination of several measures, such as self-report and drug level monitoring, to yield more accuracy has been suggested [31, 33]. Duncan et al., however, appeal for a focus on transplant outcomes rather than assessing solely medication adherence.

The current study improves upon the aforementioned shortcomings, both when it comes to the development of the intervention as well as to the methodological design. Taking the shortcomings of previous studies into consideration, the MARS-intervention is: 1) outreaching, 2)(multi) systemic, 3) theory-driven and evidence-based, 4) and tailored. The effectiveness of the intervention will be assessed in a way that takes the methodological limitations discussed into consideration. This study protocol (version 1; 6 September 2018) provides a detailed description of the design of the randomized controlled trial.

## Methods/design

This study is a single-center, parallel-group, Randomized Controlled Trial (RCT) with a baseline assessment (T0), a second assessment after a run-in period of 35 days (T1) and two follow-up assessments at the end of the intervention (T2) and 6 months thereafter (T3). Patients in the control group receive care-as-usual (CAU). Patients in the experimental group receive care-as-usual plus the MARS-intervention. The study was developed in line with the CONSORT (consolidated standards of reporting trails) and the SPIRIT (standard protocol items: recommendations for interventional trials) checklists.

### Aim and objectives

The aim of the present study is to test the effectiveness of the MARS-intervention in promoting immunosuppressive medication adherence and self-management among adolescent and adult kidney transplant recipients compared to care-as-usual (CAU).

We translated this aim into a primary research objective and several secondary objectives (Table 1).

**Table 1:** Overview of objectives and measures at different time-points

Objectives	Measures	T0	T1	T2	T3
**Primary objective**					
To test the effectiveness of the MARS- intervention (experimental group) in promoting medication adherence as measured by Electronic Monitoring in comparison to CAU (control group).	Data from Electronic Monitoring		X	X	X
**Secondary objectives patient**					
To test the effectiveness of the MARS-intervention in promoting the medication adherence as measured by secondary outcome measures in comparison to CAU (control group). To test whether the effectiveness of the MARS-intervention on medication adherence sustains after six months (T2-T3). To asses when the MARS-intervention influences medication adherence the most.	**Questionnaires patient**				
	Medication adherence (BAASIS)	X	X	X	X
	**Medical data patient file**				
	Intra patient variability (IPV)	X	X	X	X
	Rejection of the graft (treatment for rejection)			X	X
	Graft loss			X	X
	Kidney functioning (eGFR)	X	X	X	X
	**Collateral report social network**				
	Medication adherence (BAASIS)	X	X	X	X
	**Collateral report Health Care Professional**				
	Adherent to medication (Y/N)	X	X	X	X
To test the effectiveness of the MARS-intervention (experimental group) in promoting self-management in comparison to CAU (control group).	**Questionnaires patient**				
	Self-management skills (PiH-NL)	X	X	X	X
	**Medical data patient file**				
	Blood pressure	X	X	X	X
	Weight	X	X	X	X
To test the effectiveness of the MARS-intervention (experimental group) in promoting self-efficacy in comparison to CAU (control group).	**Questionnaires patient**				
	Self-efficacy (GSE)	X	X	X	X
To test the effectiveness of the MARS-intervention (experimental group) in promoting mental health in comparison to CAU (control group).	**Questionnaires patient**				
	Quality of life (WHOQoL-Bref)	X	X	X	X
	Depression & Anxiety (HADS)	X	X	X	X
	Positive & Negative affect (PANAS)	X	X	X	X
To test the effectiveness of the MARS-intervention (experimental group) in promoting social support in comparison to CAU (control group).	**Questionnaires patient**				
	Social support (MOS-SSS)	X	X	X	X
	(heiQ- subscale Social Integration and Support)	X	X	X	X
	(Self-developed item on who provides support in general and in relation to medication)	X	X	X	X
**Secondary objectives important other from the social network**					
To test the effectiveness of the MARS-intervention (experimental group) in promoting mental health among an important other in the social network of the patient in comparison to CAU (control group).	**Questionnaires important other**				
	Quality of life (CarerQol-7D)		X	X	X
	Depression (PHQ-9)		X	X	X
	Anxiety (GAD-7)		X	X	X
	Positive & Negative affect (PANAS)		X	X	X
To test the effectiveness of the MARS-intervention (experimental group) in promoting social support among an important other in the social network of the patient in comparison to CAU (control group).	**Questionnaires important other**				
	Social support (MOS-SSS)		X	X	X
	(heiQ- subscale Social Integration and Support)		X	X	X
	(self-developed item on who provides support)		X	X	X

We hypothesized that medication adherence and self-management will be higher in the MARS-intervention (experimental) group than in the control group.

### Study population

#### Patients

The study population consists of kidney transplant recipients, aged 12 years and older, with no limitation to time since transplantation. Patients are required to have a subscription of IM and have a functioning graft at the time of inclusion. Graft functioning may be deteriorating, but as long as IM is required and the patient is not expected to be on dialysis within the next 3 months they can be included. In order to avoid convenience sampling multiple ways of assessing eligibility are being used. Kidney transplant recipients are eligible for inclusion when non-adherence is signaled by either themselves (self-report), or by the nephrologist or nurse practitioner (collateral report – professional) or someone in the patient’s social network (collateral report- social network). These three indicators combined form a composite adherence score (CAS). An affirmative answer on one (or more) of these indicators is sufficient to invite the recipient to participate. A CAS is more sensitive than using a single measure of non-adherence [31, 34–36]. Furthermore, non-adherent patients who do not explicitly acknowledge their non-adherence as a problem can be identified using the CAS. Professionals form an impression of adherence through, for example, conversations with the patient, clinic attendance and blood assay monitoring. Potential participants who meet any of the following criteria will be excluded from participation: 1) patients classified as adherent on all three of the indicators forming the CAS, 2) patients on dialysis or who are expected to start dialysis within the next 3 months, 3) patients who do not understand the Dutch language, 4) patients prior to their first transplant, and 5) patients with severe psychopathology or ongoing psychological treatment. In contrast with most other studies, patients with cognitive limitations (incapacitated) are not excluded from participation because their representative(s) and social network can be involved in the intervention and give complementary information regarding problems the patient is facing.

#### Important other

Given the (multi) systemic approach of the intervention, one individual from the patients’ social network is also invited to take part in this study. The kidney transplant recipient is asked to select one person to attend (some) sessions and complete questionnaires. The patient is encouraged to choose an important other who can reflect on their medication adherence and self-management, but the patient is free to choose whomever they want. There are no limits to the criteria of this important other, other than Dutch language ability, and being over 18 years of age.

### Randomization

Patients participating in the study are randomized after a run-in period, using block randomization at the patient level, at an 1:1 allocation ratio into either one of the two groups: control group or experimental group. Prior to the start of the study the randomization list was generated by a statistician (RT) using a macro [37] in SAS Enterprise Guide 7.13 (SAS Institute Inc., Vary, NC, USA) that automates the random assignment to treatment groups. To prevent predictability of the blocked randomization, block sizes were random and hidden to the researchers and therapists. Since age of the patient can be a confounding variable, in particular being above or below the age of 18 years, age was a stratification factor. Envelopes were numbered in the same order as the randomization list and the group allocation was put in the envelopes after which they were sealed, so that these could be taken to the home of the participant and revealed accordingly.

#### Blinding

As patients need to fulfill an active role in the intervention group they cannot be blinded to the group allocation. Similarly, the person delivering the intervention cannot be blinded. On the level of the health care professional (nephrologist or nurse practitioner), we only inform the health care professional about participation in the study but not about results of randomization. However, we cannot control whether the patient discusses the intervention with their nephrologist or nurse practitioner thus blinding on the level of the healthcare professional cannot be guaranteed.

### Ethics and consent

Approval was obtained from the Institutional Review Board at the University Medical Center Rotterdam (MEC-2018-125). All participants receive written information on the study and time to consider participation. Since adolescents, adults and incapacitated patients are being included in the study, as well as important others, different participant information forms and informed consent forms are used per group. Written informed consent is obtained from both patients (≥16 years) and important others prior to participation. For patients between 12 and 15 years old, written informed consent will be obtained from both patients and the parents/guardians. From incapacitated participants, written informed consent is obtained from both patients and the legal representative.

### Procedure

**Fig. 1:**
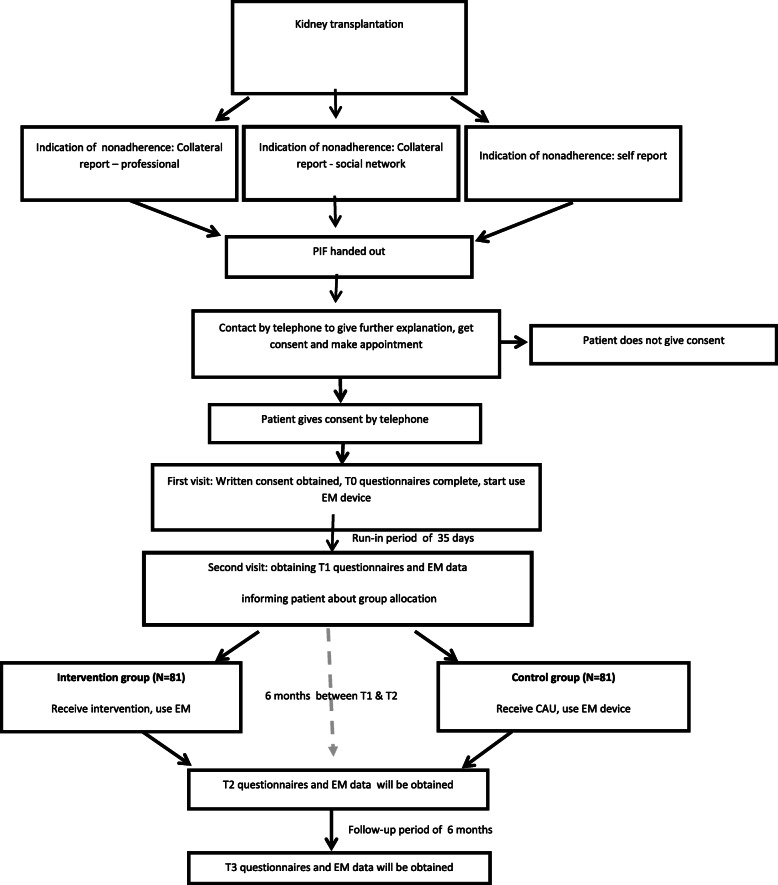
Flow diagram of study procedures

Inclusion commenced in September 2018, but inclusion is recently stopped due to Covid-19. Data collection is still ongoing. Participants are being recruited from one academic hospital in the Netherlands where they received their kidney transplant or from the local hospital after referral for post-transplant care. Patients who are eligible for the study are informed about the study by the healthcare professional at the end of their regular appointment. Thereafter the healthcare professional or someone from the research team hands out written information on the study (PIF). The healthcare professional was provided with a standardized script on introducing the study. After having time to consider participation, the researcher contacts the patients by telephone to provide additional information (when needed), answer questions, ask for consent and make an appointment to obtain written consent. This appointment can take place at the patient’s home and the important other is also invited to attend. After written consent from both parties is obtained, the first questionnaires are completed (T0) and the electronic monitoring device is explained and handed out. Since evidence suggests that adherence will be statistically higher when patients start to use the electronic monitoring device [38], a run-in period of 35 days (+/− 7 days) is incorporated. A second appointment is made to coincide with the end of the run-in period (T1), during which a second set of questionnaires is completed by patient and important other, and EM data is collected. Group assignment is revealed after questionnaires have been completed. For the following 6 months patients allocated to the experimental group receive the intervention and the control group receives CAU. Six months after T1 participants are asked to complete questionnaires and EM data is collected (T2). Subsequently, patients continue using the EM device for the follow-up period of 6 months and questionnaires are also completed at the end of this 6 months (T3) (see Fig. 1 for flow-diagram) .

#### Control group

The care-as-usual that patients of 18 years and above receive consists of regular medical check-ups in the hospital by a nephrologist or nurse practitioner (NP). The period between the check-ups varies per patient depending on for example, the time since transplantation, kidney functioning, or comorbidities. Non-adherence issues are addressed during these consultations in the outpatient clinic on indication. A NP is often involved when non-adherence is identified. Patients can be referred to a social worker or psychologist on indication. Patients aged 12–18 years old have their regular check-ups in the pediatric hospital. When problems occur, for example, problems regarding adherence, they are referred as standard to the psychologist. Family members are usually involved in the care of the adolescent.

#### Experimental group

Patients assigned to the experimental group will receive the MARS-intervention, provided by therapists specialized in working with (multi) systemic principles (DB, MR). In order to guarantee the quality of the intervention, the therapists are supervised by clinical psychologists who are also specialized in working with (multi) systemic principles (CB, JV). Peer-learning and supervision will promote fidelity to the intervention protocol.

A detailed description of the theoretical underpinning, development and content of the intervention can be found elsewhere [39]. A brief overview of the phases and operationalization of the key components of the intervention is provided here.

Five phases can be distinguished:

1. Assessment phase
2. Goal setting phase
3. Treatment phase
4. Consolidation and adjustment phase
5. Generalization and evaluation phase

During the assessment phase the focus is on building a strong relationship between patient and therapist and assessing determinants of non-adherent behavior and problems with self-management. Subsequently, important goals for the patient and the social network are assessed and divided into subgoals giving direction to the treatment phase. In this third phase strategies for change based on the specific determinants of behavior are discussed and implemented by the patient. The patient and social network are empowered to change behavior and attain their goals. During the fourth phase the emphasis is on monitoring and evaluating progress, fortifying and consolidation of new behavior whilst anticipating barriers or obstacles. Attention is given to enhancing or maintaining motivation and self-efficacy. In the fourth phase the frequency of visits is slowly reduced. Transfer of learned techniques to other situations and relapse prevention are two main themes during the final phase. Also, ending the treatment is discussed during the last phase and the intensity of visits/consultations is further reduced during this phase.

The key components that address the shortcomings of existing interventions are embedded throughout the entire intervention. The operationalization of these components is described below:

#### Outreaching

In line with an outreaching approach, the intervention is largely home-based, or at least at a location the patient prefers. Besides taking away the practical and financial barriers to travelling to the hospital, this approach also has the advantage that more members of the social network can be reached as the participant’s home is a convenient and a trusted environment.

#### (multi)systemic

At the start of the intervention an assessment of the social network of the patient takes place and the therapist assists the patient in drawing a family tree and a sociogram. These provide information on familial and social relationships that can be used to further assess who plays or can play a role in adherence and who to invite during the subsequent sessions. Depending on the aim of each session, relevant members of the social network are invited to attend. The important other (who completes the questionnaires) does not have to be present during all the sessions. In order to give the patient space to freely express him/herself, at least one session will be held with the patient alone.

#### Theory-driven and evidence-based

Besides the (multi) systemic approach the intervention is guided by several other psychological theories and evidence-based behavior change techniques. Self-efficacy, for one, is believed to be important when it comes to changing behavior [15, 40]. Therefore an important component of the intervention is raising self-efficacy and empowering the patient, through the use of techniques from solution focused (brief) therapy. The focus is on solutions and the possibilities of the patient rather than the problem. The social network is also empowered to help support the patient. Evidence-based behavior change techniques are used to change specific determinants of behavior [41, 42].

#### Tailored

As each patient is unique and the determinants of their behavior are also unique to them, the specific strategies employed to promote adherence differ per participant. In order to assess the problems experienced by the patient a visual communication aid is used, called the self-management web [43, 44]. Areas are prioritized and determinants of behavior are explored using fit circles (derived from Multi Systemic Therapy) [45]. This allows specific treatment strategies to be developed.

In addition to addressing person-specific determinants, the intervention can be tailored to the individual in other ways. The duration of the intervention can be adjusted to the goals and needs of the patient and the environment, with a maximum of 6 months. The frequency, location and duration of the appointments are tailored to the wishes of the patient and the social network. Various informants and collaborators from the social network can be involved, per participant the key players in the environment are likely to differ.

#### Administrative aspects and monitoring

Data is coded with an unique study-code per participant. The key of the code is safeguarded by the investigator and only the principal investigator and the sub-investigator will have access to this key. Data (questionnaires/interviews) will be kept for 15 years; the handling of these data will comply with the ‘Wet Bescherming Persoonsgegevens’ (Dutch Personal Data Protection). All data from the questionnaires are collected and stored in an online database, which allows for tracking any changes.

A steering committee was involved during the development of this study and remains involved during the RCT. An independent data-manager monitors the study once a year.

### Effect evaluation

#### Primary outcome measure

The primary endpoint of this study is medication adherence registered with EM, since this is referred to as the gold standard [31, 32]. A commonly used device to monitor adherence is the Medication Event Monitoring System (MEMS) bottle caps. However, the MEMS-caps have a few disadvantages such as the need to diverge from the regular Dosette box which may be confusing for patients because they have to use multiple boxes for their medications and they cannot check if they took their medication [46]. Therefore, we are using another innovative device for electronic monitoring, called the Silent Card (Adherence Innovations, Nijmegen). This silent card is being validated and negates the shortcomings of the MEMS-caps. The silent card is less invasive and disruptive for the patient since it can be used in combination with the regular Dosette box. The device can be fixed to the Dosette box and every time the immunosuppressive medication is taken, the patient needs to push a button on the card. Patients who prefer other ways of storing their medication can also use the silent card without making any alterations to their medication regimen, they simply keep the card with their medication and press the button to indicate having taken the medication. A chip registers the dates and times the button on the silent card is pressed. Data is extracted from the Silent Card using an tablet containing Near Field Communication (NFC) and Bluetooth to connect the Silent Card to the SilentReminderE app, specially developed for the Silent Card. This data is then transferred to the computer.

Medication adherence is operationalized as being adherent ≥95% as registered by the EM. We apply this stringent cut-off, as others have done, because of the clinical impact of non-adherence for this patient group [3, 30, 47]. The proportion of days adherent over the 35–215 day period (T1-T2) will be calculated. To assess whether the intervention has a sustained effect we collect EM data over the period T2-T3 (180 days). Similarly, to explore changes over time we will assess the 95% adherence rate per month as well as for the total period.

#### Secondary outcome measures patient

##### Medication adherence

In addition to EM, other secondary outcome measures are also used to assess medication adherence. This is in line with the suggestion of Schafer-Keller et al. [31] to combine several measurements to reach maximum validity. We calculate the Composite Adherence Score (CAS) combining the following measures:

- Patient self-report about non-adherence (Basel Assessment of Adherence to Immunosuppressive Medications Scale; BAASIS©-interview).
- Collateral report – important other about patients’ non-adherence using an Adjusted Basel Assessment of Adherence to Immunosuppressive Medications Scale; BAASIS©-interview.
- Collateral report – professional about patients’ non-adherence based on varying medication blood levels or behavioral indicators such as missed appointments.

The BAASIS takes both taking and timing into account, as well as drug holidays, reduction of medication doses, and persistence over the last 4 weeks. An affirmative answer on missing a dose (taking), taking IM ≥ 2 h before or after prescribed dosing time (timing), reducing the IM dose, or stopping taking medication completely (persistence) leads to being categorized as non-adherent. Patients also rate their own adherence using a visual analogue scale ranging from 0% (never take medication as prescribed) to 100% (always take medication as prescribed). The BAASIS is recommended for the use as a self-report measure among transplant recipients [1, 48]. An adjusted version of the BAASIS is used for the collateral report of the important other regarding the medication adherence of the patient. The important other is presented with the same questions as the patients on the IM adherence of the patient. Additional to the yes/no, the important other is allowed to answer I don’t know.

The collateral report from the professional is based on a yes/no answer as to whether they think a patient is adherent.

Furthermore, medical data derived from the medical records is also used to give an impression of adherence. We calculate intra patient variability (IPV) using whole blood concentrations of tacrolimus in multiple measurements over time within patients. The method previously described by Borra et al. [49] is used to make this calculation. The following transplant outcomes are collected: rejection of the graft (yes/no), graft loss (yes/no) and kidney functioning (eGFR).

##### Self-management

The 12-item Partners in Health Scale (PiH_NL) [50] is used to evaluate patients’ self-management skills. Patients can answer on a 8-point Likert scale. The PiH-NL consists out of two subscales: 1) knowledge and coping; 2)recognition plus management of symptoms, and adherence to treatment. The Cronbach’s alphas of the subscales were .80 and .72 referring to a good and acceptable internal consistency respectively. The correlation between the subscales was .43 [50].

Blood pressure and body weight are being used as clinical endpoints and are obtained from electronic patient files.

##### Self-efficacy

The Dutch adaptation of the General Self-Efficacy Scale (GSE [51]) is used to assess self-efficacy among patients. The GSE consists of 10 items. The validity of the GSE has been shown across different countries and samples [52]. The internal consistency of the GSE is good (Cronbach’s α .86 for total sample across countries, Cronbach’s α .84 for the Dutch version) [53].

##### Quality of life

Quality of life is assessed with the World Health Organization Quality of Life – Brief Version (WHOQoL-BREF [54]). The WHOQoL-BREF comprises 26 items, which form four domains: physical health, psychological health, social relationships, environment and two items representing overall quality of life and general health. Cronbach’s alphas of the domains vary between .66 and .80, with physical health having the highest internal consistency [55].

##### Mental health

The Hospital Anxiety and Depression Scale (HADS) is a 14-item scale assessing anxiety (HADS-A) and depression (HADS-D), both with seven intermingled items. The HADS was originally developed by Zigmond et al. [56], and the Dutch version has been validated among different groups of Dutch patients [57]. The most recent literature review found a mean Cronbach’s alpha of .83 and .82 for the HADS-A and HADS-D respectively [58]. The Positive and Negative Affect Schedule (PANAS [59];) comprises 20-items and is used to operationalize mental health, reflecting both negative (NA) as well as positive affect (PA). NA can be best characterized as ‘a general factor of subjective distress’ [59], PA as ‘one’s level of pleasurable engagement with the environment’ [59]. The psychometric properties of the Dutch translation of the PANAS are good (Cronbach’s alpha: PA .83, NA: .79) [60].

##### Social support

The Medical Outcomes Study Social Support Survey (MOS-SSS [61]; is used to assess perceived social support. It consists out of emotional support, tangible support, positive social interaction, affectionate support and one additional item. The MOS-SSS has excellent internal consistency, with Cronbach’s alphas ranging from .91 [61]. Since our research group translated this subscale into Dutch, it has not yet been validated. The translation was done by using forward translation (translated independently by two healthcare professionals/researchers and discussed subsequently) and blind backward translation by a native English speaker.

A subscale of the Health Education Impact Questionnaire (HEIQ [62]), Social integration and support, is also used to operationalize social support. It consists out of 5 items, which can be answered on a 4-point Likert scale. The internal consistency of the subscale is good (Cronbach’s alpha .86). We used the Dutch translation of the subscale from Been-Dahmen et al. [44].

In addition to the existing social support scales, we use a self-developed item to assess the extent to which specific persons from the social network support the patient (supplementary file 1). Furthermore, we developed one item to assess if the patient receives help from their social network when it comes to handling, thinking about and taking medication, and if so, from whom (supplementary file 1).

#### Secondary outcome measures important other

Since the patient’s social network also is involved in the intervention, the important other is also be asked to complete the following measures:

##### Mental health

To assess the quality of life of the important other we used the Care-Related Quality of Life Instrument (CarerQol) [63]. This questionnaire consists of two parts, the CarerQol-7D and the CarerQol-VAS. The CarerQol-7D consists of seven statements reflecting seven dimensions of caregiver burden. The CarerQol-VAS is a visual analogue scale ranging from 0 to 10 indicating the level of happiness a caregiver experiences. The CarerQol shows good psychometric properties among various settings [64, 65]. The Generalized Anxiety Disorder Scale-7 (GAD-7, [66]) is used to measure anxiety. This measure consists of 7 items that reflect most of the DSM-IV criteria for generalized anxiety disorder. The psychometric properties of the GAD-7 are good among primary care patients [66], the general population [67] and among a Dutch sample which received a web-based version of the GAD-7 [68]. The Patient Health Questionnaire-9 (PHQ-9 [69]) is used to assess depression. This measure consists of 9 items which represent each of the 9 DSM-IV criteria. The PHQ-9 is a validated instrument both in primary care and other medical settings [69, 70] as well as among the general population [71].

##### Social support

Social support experienced by the important other is assessed using the same instrument as the patients, namely the Medical Outcomes Study Social Support Survey (MOS-SSS). The subscale Social integration and support from the Health Education Impact Questionnaire (HEIQ) has been slightly adjusted for the use among the important other. Two out of five of the items refer to support regarding the condition, in this case the kidney transplantation. For the important other we adjusted the item to ask about support for themselves in relation to the kidney transplantation of the patient. For example, the patient item is ‘I have enough friends who help me cope with my condition’, the item for the important other is ‘I have enough friends who help me cope with his/her condition’. The remaining three items are similar to the items used among the patients.

For the important other we also use the self-developed item regarding which person(s) from their network provide the most support (supplementary file 1).

### Statistical analysis

#### Power calculation

To our knowledge there was no (multi) systemic intervention study carried out among kidney transplant recipients at the time this project commenced, making it difficult to conduct a power analysis based on a comparable study. Therefore, the power calculation is based upon a behavioral intervention study among a group of heart, liver and lung transplanted patients [72]. The intervention group showed an adherence proportion of 95.1% and the control group a proportion of 79.1%. As the MARS-intervention is more intensive than the intervention of Dobbels et al. [72] we will perform an interim analysis when half of the participants have been included. We applied a 2-sided alpha of 0.029 and a power of 0.80. Anticipating a drop-out of 10%, this led to a target of 81 participants in both groups, a total of 162.

#### Primary study parameter

The primary outcome, the proportion of participants that is adherent more than 95% of the 180 days (T1-T2) in both randomization groups as measured by EM, will be analyzed with a chi-test with continuity correction. The cut-off score of 95% is in accordance with the criteria of the Basel Assessment of Adherence to Immunosuppressive Medications Scale: BAASIS interview [48].

#### Secondary study parameters

The proportions of adherent participants based on the CAS in both randomization groups will be analyzed with a chi-test with continuity correction. The influence of biological markers on adherence will be analyzed with a logistic regression.

The continuous secondary outcomes will be analyzed with linear multi-level regression analyses. The patients form the upper level, their measures at T1 and T2 the lower level. The covariance structure will be determined with the deviance score applying restricted maximum likelihood. Treatment group, T2, the group-T2 interaction and age will be postulated as fixed effects. In case of an abnormal distribution of the scores, an appropriate transformation (logarithmic, polynomial or Blom [73]) will be applied. If no satisfying transformation can be found, the outcome will be dichotomized on the median, and a multilevel logistic regression analysis will be performed. Binary outcomes will be analyzed with multilevel logistic regression analysis, using the same levels and fixed effects as described for linear multi-level regression analysis.

To analyze whether the intervention is sustainable 6 months after the treatment, a logistic multilevel analysis will be applied including the T1, T2 and T3 measures. Adherence will be the dependent variable, and treatment group, T2, T3 and group-time interactions will be the fixed effects. The group-T2 interaction effect will present the differential intervention effect, and the T2-T3-group contrast is the effect of interest for sustainability.

To answer the question when the intervention influences the adherence most, a multi-level logistic regression analysis will be applied. The EM devices can be read every 30 days, resulting in a baseline and 6 repeated measures (at 65, 95, 125, 155, 185 and 215 days). The fixed part of the model will include treatment group, the six follow-up time points and interactions.

## Discussion

Kidney transplantation is considered the best treatment for End Stage Renal Disease. However, medium- and long-term results are being undermined because of non-adherence to the IM regimen and inadequate self-management [1]. Optimizing adherence and self-management among kidney transplant recipients has been a topic of research for several decades, but effective interventions remain scarce [20, 72]. Accordingly, our aim was to test the effectiveness of a newly developed intervention for promoting medication adherence and self-management among kidney transplant recipients; the MARS-intervention. With the present study we took into consideration shortcomings described in the literature resulting in several strengths of the current RCT. First, the MARS-intervention addresses limitations of previous interventions. It is an outreaching, (multi) systemic, theory-driven and tailored intervention. Second, chances of including a convenience sample are minimized due to the way in which patients are assessed for eligibility and the outreaching nature. Third, the current study aims to include both adolescent as well as adult participants in contrast to most other studies that focus on one or the other. Although non-adherence among adults is common, adolescence has been recognized as a period in which non-adherence is highly prevalent [14, 74] and behavior established during adolescence is predictive for behavior during adulthood [75]. Moreover, transition from pediatric to adult care has been highlighted as a risky period when it comes to adherence [76–79]. Fourth, the current study takes the social network into account, not only when it comes to participation in the intervention but also when assessing the effect of the intervention. A fifth strength of the study is the use of multiple measures of adherence. The gold standard, EM, is the primary outcome. Nevertheless, adherence is assessed in different ways as a secondary outcome allowing triangulation, which enhances reliability [12]. Furthermore, not only measures for assessing adherence are taken into account, but transplant outcomes are assessed as well. Despite the best intentions when designing this study, there are some limitations of the study. In the current study only transplanted patients were eligible for inclusion. This is mainly due to practical reasons, for example to avoid differences in measurements for assessing adherence. However, pretransplant patients are an important group, since some transplants are postponed because of non-adherence to the pretransplant regimen. Furthermore, in the current protocol the nephrologist or NP is blinded. Although this has the advantage of preventing a bias in the care-as-usual from nephrologist or NP, it also has the disadvantage of not being able to confer with the nephrologist/NP or involve them in the sessions with the patient. As the healthcare professional is an important figure in the ecological environment of the patient, it would be beneficial to involve them and their participation could be integrated into the protocol if this intervention proves to be effective. In conclusion, there is a need to improve adherence and self-management among kidney transplant recipients to optimize clinical outcomes as well as patient wellbeing. If the MARS-intervention proves to be effective, it will be a useful tool to support non-adherent transplant recipients in changing their behavior with support of the social network.

## Supplementary information

**Additional file 1: Supplementary file 1.** Self-developed social support items. Two self-developed items in addition to the existing social support scales to assess social support.

## Data Availability

The information describing the study protocol has been included within the article.

## Abbreviations

BAASIS: Basel assessment of adherence to immunosuppressive medication scale
CarerQol-7D: Care-related quality of life instrument
CAS: Composite adherence score
CAU: Care as usual
CONSORT: Consolidated standards of reporting trails
DB: Denise Beck
CB: Charlotte Boonstra
EM: Electronic monitoring
GAD-7: Generalized anxiety disorder scale-7
GSE: General self-efficacy scale
HADS: Hospital anxiety and depression scale
HeiQ: Health education impact questionnaire
IM: Immunosuppressive medication
IPV: Intra patient variability
JV: Josette Versteegh
MEMS: Medication event monitoring system
MOS-SSS: Medical outcomes study social support survey
MR: Marloes Rechards
NA: Negative affect
NFC: Near field communication
NP: Nurse practitioner
PA: Positive affect
PANAS: Positive and negative affect schedule
PHQ-9: Patient health questionnaire-9
PIF: Patient information form
PIH-NL: Partners in health scale –dutch version
RCT: Randomized controlled trial
RT: Reinier Timman
SPIRIT: Standard protocol items recommendations for interventional trials
VAS: Visual analog scale
WHO: World health organization
WHOQoL-Bref: World health organization quality of life – brief version
