# Supplementary material for: Comparison of a home-based (multi) systemic intervention to promoting Medication AdheRence and Self-management among kidney transplant recipients with care-as-usual: the MARS randomized controlled trial protocol
Source: BMC Nephrol. 2020 Aug 28;21:374. doi: 10.1186/s12882-020-02008-z (PMC7453377; doi:10.1186/s12882-020-02008-z)
Supplement: Supplementary file 1 — Additional file 1: Supplementary file 1. Self-developed social support items. Two self-developed items in addition to the existing social support scales to assess social support. [file 12882_2020_2008_MOESM1_ESM.docx]

**Self-developed social support items**

**To what extent do you feel supported by …**

|  | Never | Seldom | Sometimes | Frequently | Always | N.A. |
| --- | --- | --- | --- | --- | --- | --- |
| Significant other/  immediate family |  |  |  |  |  |  |
| Family |  |  |  |  |  |  |
| Friends |  |  |  |  |  |  |
| Other patients |  |  |  |  |  |  |
| Physicians |  |  |  |  |  |  |
| Nurses |  |  |  |  |  |  |

|  | Yes | No |
| --- | --- | --- |
| Do you get help with your medication |  |  |

If so, from whom do you get help? _______________________________________________________________________ _______________________________________________________________________
